# Preoperative cephalometric analysis to predict transoral robotic surgery exposure

**DOI:** 10.1007/s11701-014-0471-2

**Published:** 2014-06-24

**Authors:** Adam Luginbuhl, Adam Baker, Joseph Curry, Sarah Drejet, Matthew Miller, David Cognetti

**Affiliations:** 1 Department of Otolaryngology Head and Neck Surgery, Thomas Jefferson University, 925 Chestnut St, 6th Floor, Philadelphia, PA 19107 USA; 2 Department of Otolaryngology Head and Neck Surgery, University of Rochester, Rochester, NY USA

**Keywords:** Transoral robotic surgery, Cephalometrics, Exposure, Base of tongue

## Abstract

Transoral robotic surgery (TORS) is being increasingly used in the treatment of head and neck cancer and we wanted to determine the feasibility of predicting TORS access using cephalometric measurements obtained from preoperative imaging. 20 cephalometric measurements were obtained from imaging on 31 TORS base of tongue (BOT) resections and compared to adequacy of exposure. Three measurements were found to be significantly different between the restricted and adequate exposure groups. Distances from posterior pharyngeal wall (PPW) to hyoid, PPW to soft palate and epiglottis to vertical laryngeal angle were all statistically different between the two groups. Receiver operating characteristic (ROC) analysis revealed strong correlation to exposure for all three measurements with cut offs ≤30 mm between the PPW and the hyoid, ≤8.1 mm PPW and soft palate and ≥130° between the epiglottis and vertical plain of the larynx all representing restricted exposure. Duration of surgery for the restricted group, 85 min, was significantly longer than the adequate exposure group, 51 min (*p* = 0.026). Preoperative measurements of radiographic images of the oropharyngeal working space can predict restricted exposure for TORS resection of the BOT. These measures may be used in conjunction with other subjective assessment parameters to predict which patients could benefit from a staging endoscopy to determine adequate TORS exposure.

## Introduction

Transoral robotic surgery (TORS) is a technique increasingly used in surgical excision of tongue base neoplasms [1, 2]. This necessitates a greater awareness of its optimal use and limitations. One common limitation of TORS is failure to gain surgical access for adequate excision. Preoperative evaluation of each patient to determine the ability to achieve adequate exposure becomes critical, especially in light of the increased demand and limited amount of robotic availability. Despite the advantages of TORS for base of tongue (BOT) neoplasms, variations of anatomy, tumor characteristics, and surgeon expertise often contribute to the technical difficulty associated with this method and may ultimately result in aborted procedures [3]. Although a separate planning endoscopy has been suggested prior to attempting TORS [3], to date, there are no objective measurements utilized to predict the technical challenge of a TORS resection.

We propose an evaluative process to preoperatively determine the feasibility of robotic surgical access using cephalometric measurements obtained from radiographs. Van Abel proposed that preoperative evaluation of the TORS patient should include an assessment of trismus, tumor volume, tongue, degree of torus on the mandible, and whether the patient has teeth [4]. These parameters provide a global sense of the difficulty of exposure. With the addition of cephalometric measures proposed in this paper, a more informed decision can be made regarding the type of exposure the surgeon will be able to obtain in the operating room. This would minimize the need for separate planning endoscopy, allow for more informed patient counseling and facilitate treatment planning.

## Methods

**Fig. 1:**
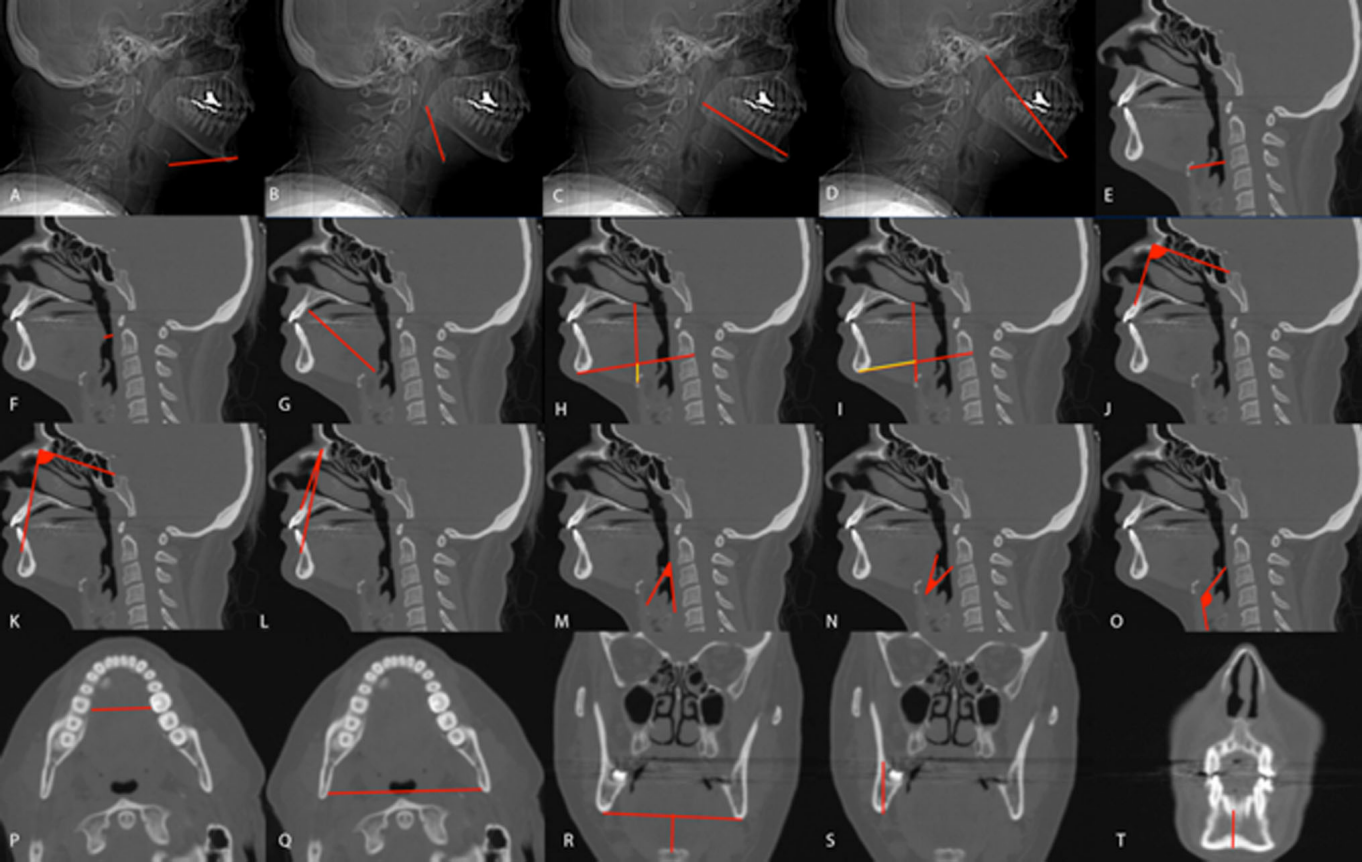
Various cephalometric measurements obtained from preoperative imaging. These measurements include: **a** hyoid to gnathion, **b** hyoid to gonion, **c** gonion to gnathion, **d** condyle to gnathion, **e** posterior pharyngeal wall to hyoid, **f** posterior pharyngeal wall to soft palate, **g** tip of tongue to vallecula, **h**, **i** two intersecting lines were created in each radiograph, one line from the gonion and to the base of vertebral body of C2, and another line from the posterior aspect of the hard palate and to the hyoid. The distance from the intersection of these two lines to the gonion and hyoid were subsequently recorded and evaluated independently and as a ratio. Angles measurements included, **j–l** measured involving the sella, nasion anterior nasal spine and suprametale, **m** measured between the epiglottis and posterior pharyngeal wall, **n** epiglottis and BOT and **o** epiglottis and larynx. Mandibular measurements included, **p**, **q** intermandubular distance at two points, **r** distance between the lower edge of the mandible and the hyoid, **s**, **t** anterior and posterior mandibular height

Between 2010 and 2012, patients who underwent attempted TORS resection at one of two tertiary care centers were included in the study. Patients met inclusion criteria if they required base of tongue (BOT) resection (*n* = 36). Five of these 36 patients were excluded due to poor documentation on ease of exposure and inadequate preoperative imaging. Institutional review board approval was obtained to perform a cephalometric analysis on this retrospective cohort. Patients who underwent an attempted resection were included regardless of whether TORS was performed. The exposure for each patient was stratified as adequate or restricted by the operative surgeon. Twenty cephalometric measurements were obtained from preoperative computed tomographs (CT) or magnetic resonance images (MRI) and compared with the ease of exposure stratification (Fig. 1). Measurements were selected based on cephalometrics used in obstructive sleep apnea literature [5]. These measurements are outlined in Fig. 1.

The mean, standard deviation, and standard error were calculated for each group and the cephalometric measurements were then compared using the *t* test. A receiver operating characteristic (ROC) curve was created for each parameter and those with a fitted ROC area greater than 0.8 were considered good predictors of a dichotomous outcome: restricted or adequate exposure.

Mallampati scores were gathered from anesthesia preoperative records and compared to the surgeon’s designation of adequate or restricted exposure using the Chi squared test. Low scores were considered a grade 1 or 2 Mallampati and grade 3 or 4 were classified as high scores. This article does not contain any studies with human or animal subjects performed by any of the authors. All procedures followed were in accordance with the ethical standards of the internal review board (IRB) and with the Helsinki Declaration of 1975.

## Results

Of the 31 patients evaluated, 15 were stratified as “adequate exposure” and 16 were deemed “restricted exposure”. Table 1 summarizes the patient’s pathology, age, operative time and body mass index (BMI). The majority of the BOT pathology results (17/31) were squamous cell carcinomas, with the remainder being lingual hypertrophy. Of the malignant lesions, T stages included T1–3. The patient’s ages varied 35–78 with BMIs of 20.7–36.1 and duration of surgery ranged 40–180 min. The duration of surgery for the restricted group, 85 min, was significantly longer than the adequate exposure group, 51 min, (*p* = 0.026).

**Table 1:** Patient demographics

Characteristic	Exposure		*p* value
	Restricted (*N* = 16)	Adequate (*N* = 15)	
Age (years)			
Median	62	54	0.40
Range	33–74	23–72	
Body mass index			
Median	29	26	0.086
Range	22–35	21–36	
Tumor stage (no.)			
T1	6	4	0.882
T2	4	8	
T3	2	0	
T4	0	0	
Lingual hypertrophy	4	3	
Duration of surgery			
Median	85	51	0.026*
Range	72–202	12–133	
Mallampati score			
Median	3	1	0.043*
Range	1–4	1–3	

* Significant *p* values

**Fig. 2:**
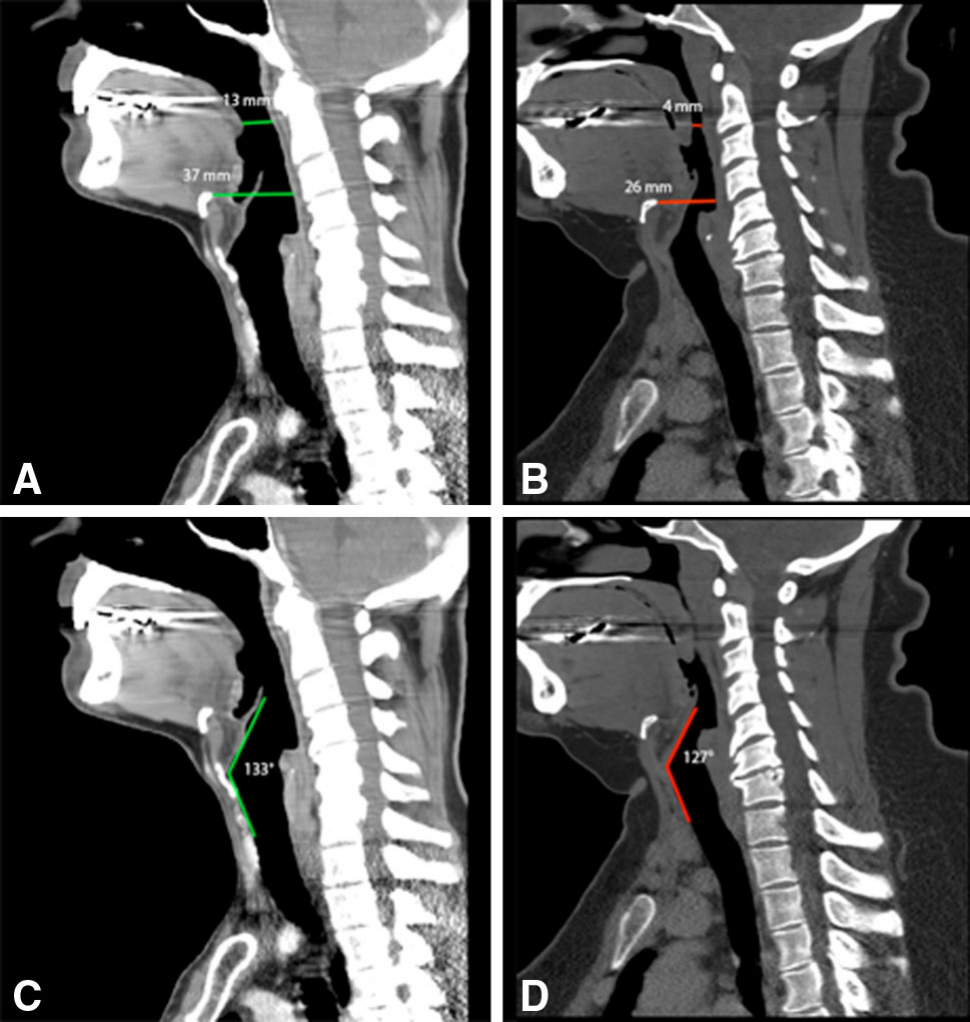
Posterior pharyngeal wall to hyoid/soft palate distance and epiglottis to vertical laryngeal angle: **a**, **c** adequate exposure, and **b**, **d** restricted exposure

Upon comparing stratifications of exposure with these measurements, 3 of the 20 measures proved to be statistically significant. The mean distance from the posterior pharyngeal wall to the hyoid in the adequate exposure group was 33.2 ± 5.6 mm, whereas the restricted group had a significantly smaller distance of 26.7 ± 5.4 mm (*p* = 0.002). In the restricted group, which has a more posteriorly situated hyoid complex, the epiglottis to vertical laryngeal angle is significantly different. The adequate exposure group had an angle of 132.9 ± 8.3° compared to 120 ± 19.9° in the restricted group (*p* = 0.04). The distance between the most inferior aspect of the soft palate as seen on the sagittal view and posterior pharyngeal wall was significantly larger in the adequate exposure group (mean of 7.8 ± 3.6 mm) when compared to the restricted group (mean of 4.7 ± 1.9 mm) (*p* = 0.006) (Fig. 2).

**Fig. 3:**
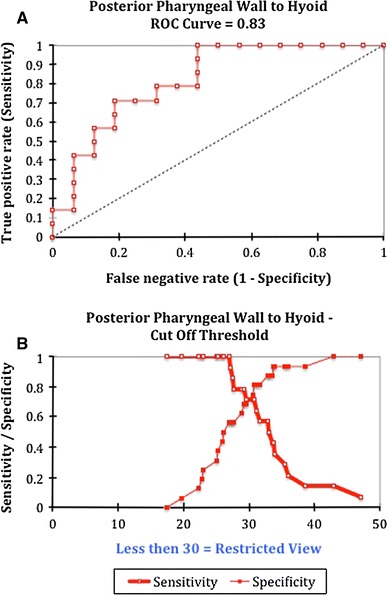
ROC curve for posterior pharyngeal wall to hyoid distance

A ROC curve was plotted to determine if the measurements could be used as a tool for selecting the cut off whereby adequacy of exposure could be predicted. When applying this statistical method to the posterior pharyngeal wall to hyoid measurement a distance less than 30 mm was the optimal number to determine if the exposure was restricted based on a plot of sensitivity/specificity vs. distance between the hyoid and posterior pharyngeal wall. The area under the curve (AUC) for this value is 0.83 with a *p* < 0.001 (Fig. 3). In restricted patients, the AUC for distance from the posterior pharyngeal wall to the soft palate is 0.80 (*p* = 0.006) with a cut off value of less than 8.1 mm and the AUC for angle of the epiglottis to larynx is 0.70 (*p* = 0.03) with a cut off value of less than 130°.

Mallampati scores for the adequate exposure group demonstrated a predominance of lower scores: 12 to 3; however, the restricted group had an almost even distribution with seven low scores and nine high scores. The correlation of the Mallampati score with difficulty of exposure was statistically significant (*p* = 0.045).

Measurements that assessed retrognathia (*p* = 0.46), mandibular height (*p* = 0.12) and tongue length (*p* = 0.63) did not significantly differ between the two groups, nor did measurements for sella nasion A (SNA) (*p* = 0.5), sella nasion B (SNB) (*p* = 0.9), or A-nasion-B (ANB) (*p* = 0.5). Length of the surgery (40–180 min) did correlate with the quality of exposure (Table 1).

## Discussion

Over the past 4 years the application of transoral robotic surgery has grown extensively, transitioning from the innovative stages to the adaptive stage with use both in academic and private practice settings. The initial reports discussed the issue of safety when considering exposure, such as assessment for retropharyngeal carotid artery and the course of the lingual artery [6, 7]. Ultimately, one of the main limiting factors in determining success of TORS is exposure. The inability to obtain exposure can lead to a cancelled TORS approach or aborting the procedure due to poor visualization or decreased confidence in the quality of resection and ultimate margin status.

The frontier of robotic surgery in head and neck surgery continues to expand. There are numerous reports in the literature regarding access to the infratemporal fossa, anterior skull base, nasopharynx, parapharyngeal space and thyroid [2, 4]. The original papers regarding TORS radical tonsillectomies and base of tongue resections also focused on feasibility and accessibility. These papers answer the question of possibility, but not of restrictions that may be faced in certain patients. Now that we know we can achieve successful operations, these data provide some insight into the limitations of access.

The clinical judgment of a restricted verses adequate exposure is subject to the evaluation of the surgeon, which may not be consistent from surgeon to surgeon. To reduce the bias of early experience all three surgeons had at least 20 TORS cases to participate in the study. We also eliminated patients that had equivocal exposure. An additional method used to minimize the judgment bias was collecting data from two institutions.

Perhaps the most critical factor in the accessibility of TORS is the location of the tumor. Isolated tonsil primaries and some of the high base of tongue primaries can be resected with less difficultly utilizing TORS. Access becomes critical when tumors extend into the vallecula, glossotonsillar sulcus and supraglottic/hypopharyngeal regions. In order to isolate a homogeneous group for analysis in this study we included only those patients with base of tongue pathology. We cannot directly extrapolate these data to supraglottic or hypopharyngeal tumors, but by inference we believe access to these areas would be equally restrictive or open.

The patient selection process could be streamlined by clinically and radiographically identifying those patients that may pose an access challenge and schedule them for a staging panendoscopy. Those patients that preoperatively appear to have easier access could be directly scheduled for robotic resection. The measurements determined in this study using ROC curve and *t* test analysis would indicate that a posterior pharyngeal wall to hyoid measurement less than 30 mm is considered a risk factor for a restricted view. Similarly a distance less than 8.1 mm from the posterior pharyngeal wall to the soft palate and an angle less than 130° between the epiglottis and the larynx would also indicate a possibly challenging exposure.

Since the hyoid bone is the tethering point of the intrinsic musculature of the tongue, we anticipated finding significance with respect to the length of the tongue and the relationship of the hyoid bone to the mandible. These measurements, however, did not compute out to determine the difficulty of exposure. However, a lower Mallampati score was predictive of adequate exposure. This score measures the height of the tongue base in relationship to palate and this measurement has been correlated with the Cormack–Lehane classification system which focuses on the view seen on direct laryngoscopy [8, 9]. During the preoperative setting the surgeon can make an informed decision regarding exposure difficulty based on the objective measurement criteria outlined in these data along with, the amount of trismus, presence or absence of teeth or mandibular tori and tumor location.

## Conclusions

Preoperative measurements of radiographic images of the oropharyngeal working space determined that a distance less than 8 mm from the posterior pharyngeal wall to the soft palate and/or 30 mm from the posterior pharyngeal wall to the hyoid, and/or an angle less than 130° between the epiglottis and larynx, may represent restricted exposure for TORS resection of the BOT. These measures may predict which patients would benefit from a staging endoscopy to determine adequate TORS exposure.
